# Supplementary material for: Modeling glioblastoma heterogeneity as a dynamic network of cell states
Source: Mol Syst Biol. 2021 Sep 16;17(9):e10105. doi: 10.15252/msb.202010105 (PMC8444284; doi:10.15252/msb.202010105)
Supplement: Supplementary file 6 — Source Data for Figure 5 [file MSB-17-e10105-s004.zip › Figure5A_sourcedata/GSEA_3071/hallmarks_stateVI.GseaPreranked.1623742557031/HALLMARK_INTERFERON_GAMMA_RESPONSE.html]

Details for gene set HALLMARK\_INTERFERON\_GAMMA\_RESPONSE[GSEA]

|  || Dataset | state63071 |
| Phenotype | NoPhenotypeAvailable |
| Upregulated in class | na\_pos |
| GeneSet | HALLMARK\_INTERFERON\_GAMMA\_RESPONSE |
| Enrichment Score (ES) | 0.22814047 |
| Normalized Enrichment Score (NES) | 1.365055 |
| Nominal p-value | 0.11640212 |
| FDR q-value | 0.14636177 |
| FWER p-Value | 0.589 |
Table: GSEA Results Summary

  

Fig 1: Enrichment plot: HALLMARK\_INTERFERON\_GAMMA\_RESPONSE      
 Profile of the Running ES Score & Positions of GeneSet Members on the Rank Ordered List

  

| PROBE | GENE SYMBOL | GENE\_TITLE | RANK IN GENE LIST | RANK METRIC SCORE | RUNNING ES | CORE ENRICHMENT || 1 | MT2A |  |  | 58 | 0.859 | 0.0297 | Yes |
| 2 | CD274 |  |  | 64 | 0.835 | 0.0729 | Yes |
| 3 | UPP1 |  |  | 218 | 0.590 | 0.0618 | Yes |
| 4 | TNFAIP2 |  |  | 244 | 0.568 | 0.0852 | Yes |
| 5 | RIPK2 |  |  | 267 | 0.555 | 0.1087 | Yes |
| 6 | TXNIP |  |  | 402 | 0.487 | 0.0975 | Yes |
| 7 | TNFAIP3 |  |  | 445 | 0.473 | 0.1111 | Yes |
| 8 | PFKP |  |  | 473 | 0.463 | 0.1283 | Yes |
| 9 | SOCS1 |  |  | 489 | 0.458 | 0.1485 | Yes |
| 10 | PIM1 |  |  | 582 | 0.430 | 0.1459 | Yes |
| 11 | PNP |  |  | 625 | 0.418 | 0.1566 | Yes |
| 12 | TNFSF10 |  |  | 631 | 0.416 | 0.1774 | Yes |
| 13 | MVP |  |  | 642 | 0.413 | 0.1967 | Yes |
| 14 | ARL4A |  |  | 704 | 0.395 | 0.2008 | Yes |
| 15 | IRF1 |  |  | 771 | 0.382 | 0.2028 | Yes |
| 16 | SOCS3 |  |  | 775 | 0.381 | 0.2223 | Yes |
| 17 | PML |  |  | 874 | 0.365 | 0.2145 | Yes |
| 18 | EPSTI1 |  |  | 955 | 0.352 | 0.2111 | Yes |
| 19 | PSME2 |  |  | 962 | 0.351 | 0.2281 | Yes |
| 20 | PTPN2 |  |  | 1044 | 0.338 | 0.2237 | No |
| 21 | PSMB2 |  |  | 1171 | 0.320 | 0.2057 | No |
| 22 | IL15 |  |  | 1353 | 0.297 | 0.1712 | No |
| 23 | HIF1A |  |  | 1458 | 0.284 | 0.1574 | No |
| 24 | IFI44L |  |  | 1742 | 0.256 | 0.0924 | No |
| 25 | ISG20 |  |  | 1756 | 0.255 | 0.1024 | No |
| 26 | TAPBP |  |  | 1817 | -0.252 | 0.0991 | No |
| 27 | STAT3 |  |  | 1861 | -0.259 | 0.1010 | No |
| 28 | ADAR |  |  | 1912 | -0.265 | 0.1013 | No |
| 29 | PARP14 |  |  | 2145 | -0.304 | 0.0530 | No |
| 30 | ARID5B |  |  | 2156 | -0.306 | 0.0665 | No |
| 31 | STAT1 |  |  | 2199 | -0.314 | 0.0716 | No |
| 32 | STAT2 |  |  | 2345 | -0.342 | 0.0495 | No |
| 33 | RNF213 |  |  | 2383 | -0.350 | 0.0579 | No |
| 34 | C1R |  |  | 2388 | -0.351 | 0.0755 | No |
| 35 | B2M |  |  | 2460 | -0.369 | 0.0754 | No |
| 36 | CASP3 |  |  | 2699 | -0.426 | 0.0320 | No |
| 37 | ST8SIA4 |  |  | 2719 | -0.430 | 0.0496 | No |
| 38 | AUTS2 |  |  | 2747 | -0.439 | 0.0656 | No |
| 39 | IFITM3 |  |  | 2851 | -0.477 | 0.0624 | No |
| 40 | PDE4B |  |  | 2948 | -0.513 | 0.0630 | No |
| 41 | LGALS3BP |  |  | 2989 | -0.533 | 0.0804 | No |
| 42 | TNFAIP6 |  |  | 3085 | -0.578 | 0.0848 | No |
| 43 | ST3GAL5 |  |  | 3172 | -0.639 | 0.0950 | No |
| 44 | SRI |  |  | 3179 | -0.643 | 0.1277 | No |
Table: GSEA details [plain text format]

  

Fig 2: HALLMARK\_INTERFERON\_GAMMA\_RESPONSE: Random ES distribution      
 Gene set null distribution of ES for **HALLMARK\_INTERFERON\_GAMMA\_RESPONSE**
